# PREDICT-H Protocol: A Multicenter Prospective Cohort Study on Preoperative Anatomical Determinants and Postoperative Complications in Primary Hypospadias Repair

**DOI:** 10.3390/diagnostics15162087

**Published:** 2025-08-20

**Authors:** Tariq Abbas

**Affiliations:** 1 Urology Division, Department of Surgery, Sidra Medicine, Doha P.O. Box 26999, Qatar; tabbas@sidra.org or tariq2c@hotmail.com; 2 College of Medicine, Qatar University, Doha P.O. Box 2713, Qatar; 3 Weill Cornell Medicine—Qatar, Doha P.O. Box 24144, Qatar

**Keywords:** hypospadias, pediatric urology, congenital anomalies, surgical outcomes, anatomical predictors, prospective cohort study, risk stratification, nomogram, complications, multicenter study

## Abstract

**Background**: Hypospadias is a common congenital anomaly in boys, marked by ectopic urethral meatus and a wide range of anatomical variants such as chordee and atypical glans morphology. Despite advancements in surgical techniques, complication rates remain high and unpredictable due to heterogeneity in anatomy and a lack of standardized preoperative assessments. Retrospective studies suggest associations between specific anatomical features and postoperative complications; however, high-quality prospective, multicenter evidence is currently lacking. **Methods**: The PREDICT-H (Prospective Research on Essential Determinants Influencing Complication Trends in Hypospadias) study is a multicenter, prospective cohort study aiming to enroll approximately 1450 boys aged 1–12 years undergoing primary hypospadias repair at ten or more tertiary pediatric urology centers. A standardized preoperative assessment protocol will document detailed anatomical parameters, including urethral plate width and length, glans size, meatal location, chordee severity, and GMS score. Intraoperative variables and surgical techniques will be recorded. Postoperative outcomes, including urethrocutaneous fistula, meatal stenosis, and recurrent chordee, will be assessed at ≥6 months follow-up. Statistical analyses will include multivariate logistic regression and advanced modeling to identify independent predictors and develop a validated risk prediction nomogram. Interobserver reliability of anatomical assessments will also be evaluated. **Results**: As this is a study protocol, results are not yet available. Data collection is ongoing and will be analyzed upon completion of the planned follow-up period. The primary outcome will be the incidence of postoperative complications and the development of a predictive nomogram for individualized risk estimation. **Conclusions**: The PREDICT-H study is designed to provide robust, prospective evidence on the anatomical determinants of postoperative complications in hypospadias surgery. The development of a validated, clinically applicable risk prediction tool could standardize preoperative assessment and enhance individualized surgical planning. Findings from this study are expected to support evidence-based practice and inform future clinical guidelines.

## 1. Introduction

Hypospadias is one of the most common congenital anomalies in males, occurring in approximately 1 in 200 to 300 live births. It is characterized by an ectopic urethral meatus located on the ventral aspect of the penis, often accompanied by a ventrally deficient foreskin and varying degrees of chordee (ventral curvature). The condition presents a wide anatomical spectrum—from distal forms near the glans to severe proximal variants involving significant curvature and scrotal transposition—each of which may demand different surgical strategies and pose different risks for postoperative complications [1].

Surgical repair is typically performed during early childhood, with primary goals of achieving a functionally straight penis with a terminal meatus and ensuring acceptable cosmetic and psychosocial outcomes. Despite significant advancements in operative techniques—including the widespread adoption of the tubularized incised plate (TIP) urethroplasty and various flap or graft-based reconstructions—postoperative complications remain frequent [2]. Reported complication rates range from 10% to over 40%, depending on the severity of the anomaly, the surgical method used, and the surgeon’s experience. Common complications include urethrocutaneous fistula, meatal stenosis, urethral stricture, residual chordee, and the need for revision surgery.

A central challenge in hypospadias surgery is the inability to reliably predict which patients are at higher risk for complications. Over the past two decades, numerous cohort studies have attempted to identify anatomical factors associated with poorer outcomes. Variables such as urethral plate width [3] and quality [4], glans diameter [5], meatal location [6], and severity of chordee [7] have repeatedly been suggested as potential predictors. However, most of this evidence stems from small, single-center studies with heterogeneous methodologies and inconsistent definitions. There is also a lack of consensus regarding how best to quantify these anatomical parameters or how they should influence surgical planning. Compounding this problem is the absence of standardized preoperative assessment protocols, which has led to widespread variability in measurement techniques and subjective assessments. Efforts to create composite scoring systems—such as the Glans–Meatus–Shaft (GMS) score [7]—have shown promise but have not been universally adopted or validated in large, prospective settings. Additionally, little is known about the interobserver reliability of anatomical assessments, even among experienced pediatric urologists [8].

Despite the well-recognized need for improved preoperative risk stratification, no large-scale, multicenter, prospective study has systematically evaluated anatomical predictors of surgical outcomes in hypospadias. To date, there is no validated nomogram or prediction tool that integrates anatomical and surgical variables to estimate complication risks in individual patients. This lack of predictive modeling limits the ability of surgeons to personalize care, choose the most appropriate surgical technique, set realistic expectations with families, or select patients for closer postoperative monitoring [9].

The **PREDICT-H study** (Prospective Research on Essential Determinants Influencing Complication Trends in Hypospadias) is designed to address these critical gaps. It is the first prospective, multicenter cohort study powered to evaluate the association between standardized, preoperatively assessed anatomical features and postoperative complications in primary hypospadias repair. By collecting high-quality, granular data across a large, diverse patient population and by applying modern statistical modeling techniques, this study aims to develop and internally validate a clinically useful nomogram to predict postoperative complication risks. In doing so, it seeks to enable evidence-based, individualized surgical planning and improve both short- and long-term outcomes for patients with hypospadias.

## 2. Methods

### 2.1. Study Design

The PREDICT-H study is a multicenter, prospective, observational cohort study involving at least ten tertiary-care pediatric urology centers. Patient recruitment will occur over 24 months, with a minimum follow-up duration of 6 months per patient. An extended follow-up to 12 months will be optional at participating sites.

### 2.2. Sample Size

A total sample size of approximately 1450 patients is anticipated. This number was derived based on an estimated 20% complication rate, 14 predictive variables in the final multivariable model, and requirements for at least 15 outcome events per predictor to avoid overfitting. An additional 15% was added for anticipated loss to follow-up, and 10% was added to account for potential clustering effects across centers.

### 2.3. Objectives

The **primary objective** of the PREDICT-H study is to prospectively evaluate the predictive value of specific preoperative anatomical variables on postoperative complication rates in boys undergoing primary hypospadias repair. To achieve this, this study will enroll a large multicenter cohort and apply a standardized, detailed preoperative assessment protocol.

The **secondary objectives** include the development and internal validation of a predictive nomogram for surgical complications by integrating anatomical and clinical variables. Additionally, this study aims to analyze how different surgical techniques may act as confounders or effect modifiers to influence outcomes. Another key goal is to assess the interobserver reliability of anatomical measurements to ensure reproducibility and consistency across centers. Finally, this study seeks to establish a standardized anatomical assessment protocol that can be used reliably in both clinical practice and future research. Nomogram development will primarily rely on anatomical and surgical variables; however, known clinical variables such as endocrine abnormalities or karyotype findings will also be considered if adequately reported and recorded across sites.

### 2.4. Study Population

#### 2.4.1. Inclusion Criteria

- Male patients aged 1 to 12 years.
- Diagnosis of hypospadias (distal, midshaft, or proximal).
- Scheduled for primary surgical repair.
- Informed consent obtained from a parent or legal guardian.

#### 2.4.2. Exclusion Criteria

- Previous hypospadias or penile surgery.
- “Major associated genital anomalies such as ambiguous genitalia or confirmed disorders of sexual development (DSD), including cases with syndromic DSD, severe penoscrotal transposition, or atypical genitalia requiring endocrine/genetic workup. Isolated micropenis without syndromic features will not be excluded”. Inability to comply with follow-up or refusal to consent.
- “Patients with isolated extragenital malformations (e.g., renal or cardiac anomalies) will not be excluded unless the condition has a direct impact on genital development or surgical outcome”.

### 2.5. Preoperative Assessment

#### 2.5.1. Standardization and Training

Participating centers will receive a comprehensive manual including pictorial guides and written instructions on measurement techniques. Training sessions will ensure consistency in data collection, with calibration exercises conducted biannually. Measurement reliability will be assessed using intra-class correlation coefficients (ICCs) and Cohen’s kappa.

#### 2.5.2. Anatomical Variables Collected

To ensure standardized, reproducible data across all participating centers, the following key anatomical variables will be assessed using pre-established protocols, with dual-observer measurements and calibration procedures to enhance inter-rater reliability. “Where available, relevant clinical variables such as endocrine disorders (e.g., disorders of androgen synthesis or action) and genetic diagnoses (e.g., karyotype abnormalities) will also be included in the multivariable models to assess their potential contribution to postoperative outcomes”.

##### Meatal Location

Visual inspection and photographic documentation will be used to categorize the location of the urethral meatus into six standard zones: glanular, coronal, distal penile, midpenile, proximal penile, and scrotal/perineal areas. The online *Birth Defect Surveillance* manual by the Centers for Disease Control and Prevention (CDC) features a hypospadias atlas that provides photographic representations of various phenotypic subtypes of hypospadias.

##### Urethral Plate Length

**Figure 1:**
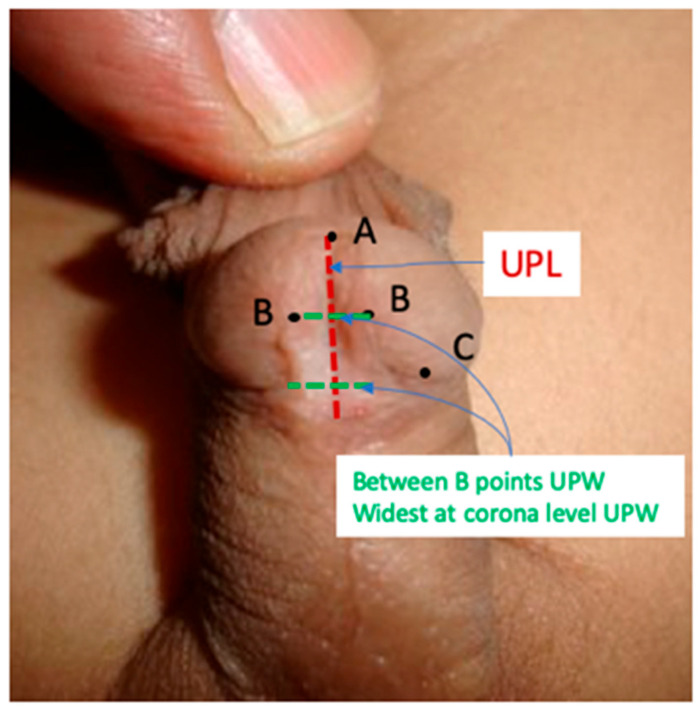
Standardized measurement of urethral plate length (UPL) and urethral plate width (UPW). Point A marks the distal extent of the mucocutaneous junction in the midline of the urethral plate; point B corresponds to the level of the glanular knobs; and point C identifies the coronal territory of the glans where the glans changes its direction, delineating the proximal boundary of the glans wings.

Urethral plate length (UPL) is measured from the hypospadiac meatus to the tip of the glans along the midline of the urethral plate using sterile digital calipers or fine rulers (Figure 1).

##### Urethral Plate Width

Urethral plate width (UPW) is measured at the widest point of the urethral plate and between the two “B points” (medial borders of the plate) using sterile digital calipers or fine rulers (Figure 1).

##### Urethral Plate Depth/Configuration

Evaluation is performed via a visual assessment of plate depth as flat, shallow, or deep.

##### Glans Width (GW)

**Figure 2:**
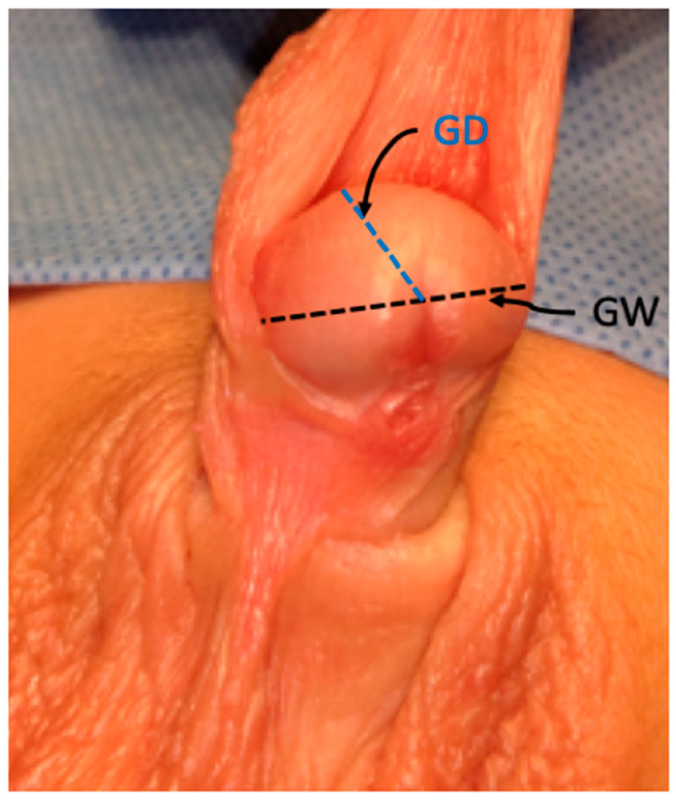
Standardized measurement of glans width (GW) and glans width (GD).

Using digital calipers, the transverse diameter of the glans is measured at its widest horizontal point, anterior to the corona. This measurement is performed under anesthesia, in a relaxed state, and before surgical manipulation. Two measurements are obtained by each of two independent evaluators (Figure 2).

##### Glans Depth (GD)

Anteroposterior depth is evaluated at the glans midline using calipers, using two evaluators, and measurements are performed in two replicates (Figure 2).

##### GMS Score (Glans–Meatus–Shaft)

This is a composite score evaluating three domains: **G (Glans size/configuration)**, **M (Meatal location),** and **S (Shaft curvature).** Each component is scored from 1 (least favorable) to 3 or 4 (most favorable), yielding a total score range from 3 to 12 (Table 1).

##### Penile Curvature Severity

**Figure 3:**
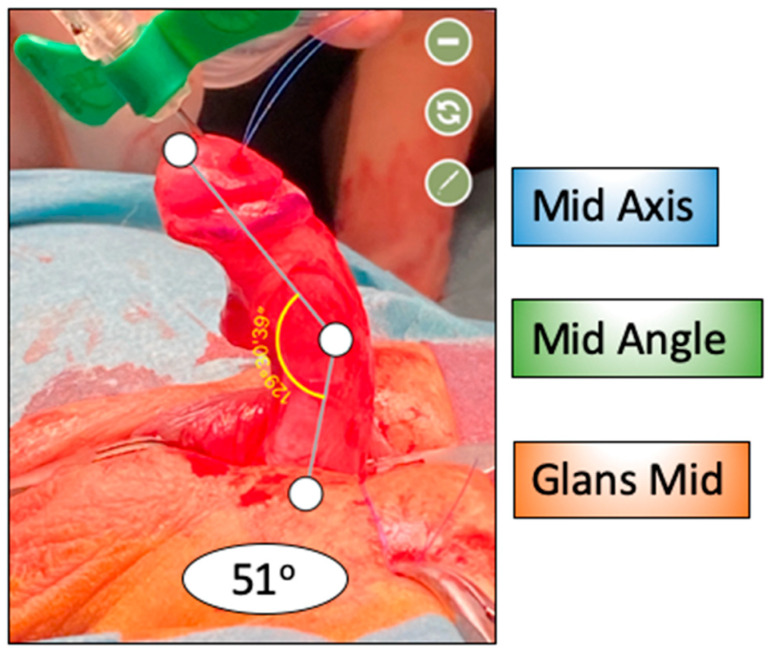
Standardized measurement of the degree of penile curvature.

The artificial erection test was performed intraoperatively using saline or prostaglandin injections. Angle of penile curvature was measured using a sterile goniometer or validated mobile applications like the Angle Meter 360 (by ALEXEY KOZLOV) (Figure 3).

##### Stretched Penile Length—SPL

**Figure 4:**
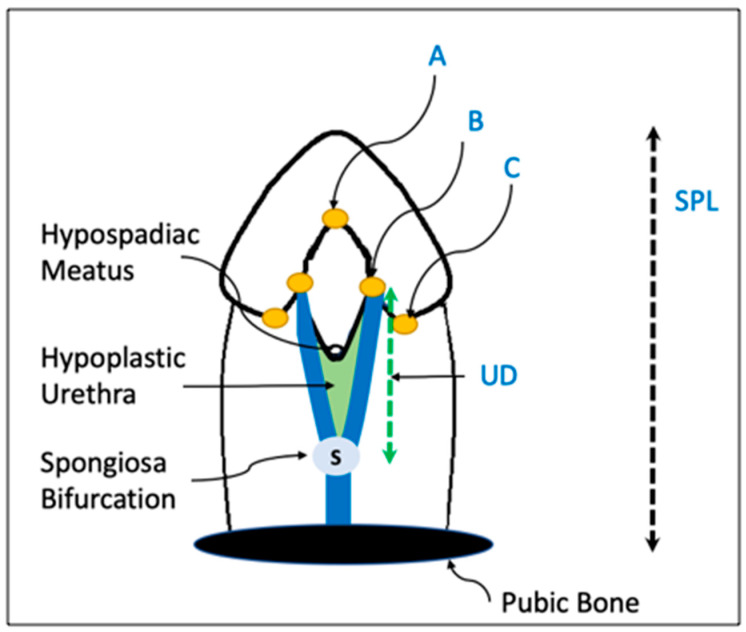
Standardized measurements of stretched penile length (SPL) and urethral defect (UD). Point A marks the distal extent of the mucocutaneous junction in the midline of the urethral plate; point B corresponds to the level of the glanular knobs; and point C identifies the coronal territory of the glans where the glans changes its direction, delineating the proximal boundary of the glans wings. (S) spongiosa bifurcation point.

With the penis fully stretched from the pubic bone to the glans tip in the supine position, the length is measured using a rigid ruler. Measurements are standardized to avoid excessive force; pubic fat pad compression is standardized across sites (Figure 4).

##### Urethral Defect Length (UDL)

After complete penile degloving, the “B points” (representing the lateral borders of the urethral plate at the junction with the corpora) can be identified. The distal reference point is the point of maximum plate narrowing at the glans or the distal B point. The proximal reference point is the bifurcation of the corpus spongiosum, marking the native urethral end. A sterile ruler or digital calipers are used to measure the distance along the midline between these two points (Figure 4).

##### Anogenital Distance (AGD)

AGD is the distance from the center of the anus to the base of the scrotum and is a sensitive marker of genital development and fetal androgen exposure. The child is placed supine with their knees flexed and hips abducted (frog-leg position) to fully expose the perineum. Using a rigid ruler, the distance is measured from the posterior reference point and the center of the anal verge to the Anterior reference point and the posterior-most visible point of the scrotal base or penoscrotal junction. Measurements are taken before the surgical field is prepped to avoid tissue distortion.

##### Plate Objective Scoring Tool (POST)

**Figure 5:**
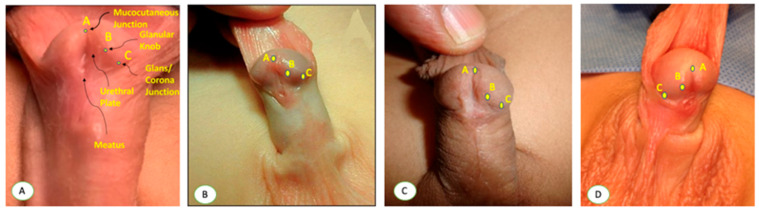
(**A**) Determination of the three key anatomical landmarks used in hypospadias evaluation: point A marks the distal extent of the mucocutaneous junction in the midline of the urethral plate; point B corresponds to the level of the glanular knobs; and point C identifies the coronal territory of the glans where the glans changes its direction, delineating the proximal boundary of the glans wings. (**B**–**D**) Other examples.

A quantitative scoring tool assesses urethral plate quality and shape using a visual exam and photo documentation (Figure 5).

##### Urethral Plate-to-Glans Width Ratio (U/G Ratio)

This is calculated from UPW and GW measurements.

##### Urethral Defect Length/SPL

This is calculated from UDL and SPL measurements (Table 2).

### 2.6. Intraoperative Documentation

Surgical details include the technique used (e.g., TIP, Mathieu, staged, and Duckett), use and duration of stents or catheters, tissue coverage techniques (e.g., dartos or spongioplasty), graft source (e.g., preputial, buccal, or scrotal), and intraoperative complications. Surgeon experience (years in practice and hypospadias case volume) will be documented to explore its impact on outcomes.

### 2.7. Postoperative Follow-Up

Patients will be evaluated at 1, 3, and 6 months postoperatively, with an optional 12-month visit for long-term outcomes. Each visit will include a clinical examination for complications such as fistula, stenosis, or recurrent chordee. Cosmetic outcomes will be assessed using validated tools like the Pediatric Penile Perception Score (PPPS), and urinary stream will be assessed subjectively and via uroflowmetry in toilet-trained children. Standardized photography will be conducted on each visit.

### 2.8. Outcomes to Be Measured

#### 2.8.1. Primary Outcome

The primary outcome is the composite postoperative complication rate at 6 months, which includes the following:

- **Meatal Stenosis**: Narrowing of the external urethral meatus associated with obstructive voiding symptoms (e.g., straining, prolonged voiding, or urinary retention). Confirmed by meatal calibration <8 French (Fr) before puberty.
- **Urethral Stricture**: Fixed narrowing of the urethral lumen due to fibrotic scar tissue, presenting with obstructive symptoms (weak stream and retention), and confirmed on urethroscopy.
- **Urethral Diverticulum**: A segmental, sac-like outpouching of the urethral wall observed during voiding, often presenting as post-void dribbling or swelling, and confirmed via dynamic imaging or endoscopy.
- **Urethrocutaneous Fistula**: An abnormal communication between the urethral lumen and the skin, manifesting as urine leakage through a secondary opening. Categorized by a **small fistula** size ≤3 mm and **large fistula** size >3 mm.
- **Urinary Tract Infection (UTI)**: Defined per the American Academy of Pediatrics (AAP) criteria. **Febrile UTI**: positive urine culture with fever ≥38 °C; **Afebrile UTI**: positive culture with pyuria but without systemic signs
- **Glans Dehiscence**: Complete separation of the glans wings.
- **Recurrent Penile Curvature**: Residual or recurrent penile curvature >30° (ventral or lateral), objectively assessed during pharmacological or artificial erection.
- **Penile Torsion**: Rotation of the penile shaft is identified when the glans deviates >30° from the vertical axis. Direction specified: **clockwise** or **counterclockwise**.

#### 2.8.2. Secondary Outcomes:

- Cosmetic and functional outcomes of the Hypospadias Objective Penile Evaluation (HOPE) [10] and Hypospadias Objective Scoring Evaluation (HOSE) [11].
- Time to complications.
- Association between specific anatomical variables and individual complications.

### 2.9. Data Management

Data will be entered into a centralized, secure, web-based platform with access controls. Regular data audits will be performed, and automatic validation rules will ensure data completeness. Patient identifiers will be encrypted and stored separately from clinical data. Photographs will be securely uploaded and linked to de-identified patient IDs.

### 2.10. Statistical Analysis

Descriptive statistics will summarize the cohort’s baseline characteristics. Measurement reliability will be assessed using ICCs and Cohen’s kappa. Univariate analysis will explore associations between individual anatomical features and complications using chi-square tests for categorical variables and t-tests or Mann–Whitney U tests for continuous variables. Multivariable logistic regression models will be developed using variables identified from univariate analysis and clinical judgment. Model performance will be assessed through discrimination using the area under the ROC curve (AUC), calibration using the Hosmer–Lemeshow test and calibration plots, and internal validation using Bootstrap resampling (1000 iterations). A predictive nomogram will be constructed based on the final model. Subgroup analyses will explore the impact of hypospadias severity and surgical technique.

## 3. Ethics and Dissemination

### 3.1. Ethical and Safety Considerations

The PREDICT-H study protocol has been reviewed and approved by the institutional review boards (IRBs) or equivalent ethics committees at the main center, Sidra Medicine SDR 600058, IRB 1527961. Each site will obtain local ethical clearance prior to initiating patient recruitment. The study will be conducted in accordance with the principles outlined in the Declaration of Helsinki and Good Clinical Practice (GCP) guidelines. Written informed consent will be obtained from the parents or legal guardians of all participants prior to enrollment. Age-appropriate assent will also be sought where applicable, depending on local regulations and the child’s level of understanding. Participation is entirely voluntary, and families will have the right to withdraw at any time without affecting the clinical care of the patient.

No experimental procedures or deviations from standard surgical practice will be introduced in the study protocol. All data collected will pertain to standard clinical assessments or routinely performed intraoperative measurements, minimizing risk to participants. This study does not involve the administration of investigational drugs or devices. To ensure patient confidentiality, all study data will be de-identified and stored on secure, password-protected electronic data capture systems. Access to identifiable data will be restricted to site-specific investigators. Data sharing agreements and secure cloud-based platforms will be employed to ensure the safe transfer and storage of data across collaborating sites. Regular data audits will be conducted to ensure protocol adherence, completeness, and accuracy.

### 3.2. Dissemination Plan

The findings of the PREDICT-H study will be disseminated through peer-reviewed scientific journals, conference presentations at national and international pediatric urology meetings, and stakeholder briefings with surgical societies and training bodies. The predictive nomogram developed through this study will be made publicly accessible through an online interface and a clinical app, subject to validation and appropriate licensing. Educational materials summarizing the results will be prepared for distribution among patients, families, and advocacy groups in understandable language to support shared decision-making.

A data availability statement and metadata will accompany the dataset to promote reuse and secondary analyses by the broader scientific community. Requests for extended data access or collaboration will be considered upon submission of a written proposal to the study’s steering committee.

## 4. Results

As the PREDICT-H study is in the preparatory and early recruitment phase, no clinical or outcome data are yet available for analysis. Recruitment across the participating tertiary pediatric urology centers is scheduled to commence in October 2025, with an anticipated enrollment period of approximately 24 months. The study team has completed training and calibration exercises to ensure standardized anatomical measurements and data collection procedures are uniformly implemented at all sites. A centralized, secure, web-based database has been established and tested for data entry, quality control, and management. Ongoing monitoring plans are in place to assess the recruitment progress, data completeness, and adherence to protocol standards. Interim analyses will be conducted after reaching predefined enrollment milestones to evaluate data quality, interobserver reliability, and early trends in complication rates. The final results and predictive modeling outcomes will be reported upon completion of the follow-up period for all enrolled patients.

## 5. Discussion

The PREDICT-H study represents a pioneering prospective, multicenter cohort investigation into the influence of preoperative anatomical variables on postoperative outcomes following primary hypospadias repair. By rigorously standardizing the assessment of key anatomical features such as urethral plate width and length, glans size, chordee severity, and meatal location across multiple high-volume pediatric urology centers, this study seeks to overcome longstanding limitations in hypospadias research caused by heterogeneous measurement methods and retrospective study designs. The ultimate aim is to develop and internally validate a clinically useful nomogram that predicts individual complication risks, thereby advancing personalized surgical planning and patient counseling.

Although this protocol manuscript precedes the study results, its rationale and design are grounded in extensive prior research highlighting the variability in hypospadias anatomy and its suspected impact on surgical outcomes. Previous meta-analyses and retrospective cohort studies have identified urethral plate width, glans size, severity of chordee, and meatal position as potential predictors of postoperative complications; yet, findings have often been inconsistent and limited by small sample sizes, single-center biases, and a lack of standardized measurement protocols.

Unlike earlier studies, PREDICT-H’s prospective and multicenter design enhances the generalizability of findings across diverse patient populations and surgical practices. Furthermore, the planned rigorous assessment of interobserver reliability addresses a critical gap in the literature, as prior reports have seldom quantified measurement reproducibility, which is a key factor for clinical applicability. Comprehensive documentation of surgical techniques and intraoperative factors also allows for nuanced analysis of confounding or effect-modifying influences, which have been underexplored in previous investigations.

In contrast to existing scoring systems such as the GMS score or other composite indices, PREDICT-H aims to integrate anatomical, clinical, and surgical variables into a validated predictive nomogram. This tool could provide surgeons with practical, evidence-based means to estimate individualized complication risk preoperatively, which is an advancement that may significantly improve decision-making and family counseling. “The inclusion of patients with extragenital malformations, while enhancing generalizability, may introduce confounding effects on postoperative outcomes and represents a potential limitation”.

The PREDICT-H study addresses a critical unmet need in hypospadias surgery by prospectively elucidating the relationship between objective anatomical parameters and postoperative complication risks in a large, multicenter cohort. Its comprehensive and standardized approach promises to produce the first validated predictive nomogram, facilitating personalized surgical planning and improved patient and family counseling. By establishing a robust, reproducible anatomical assessment protocol, PREDICT-H also lays the foundation for future research and clinical quality improvement. Ultimately, this work has the potential to significantly enhance outcomes and satisfaction in boys undergoing hypospadias repair worldwide.

## Figures and Tables

**Table 1:** Scoring system of the GMS.

**Glans (G) Score**	**Description**
1	Good glans size; healthy urethral plate, deeply grooved
2	Adequate glans size; adequate urethral plate, grooved
3	Small glans size; urethral plate narrow, with some fibrosis or flat areas
4	Very small glans; urethral plate indistinct, very narrow, or flat
**Meatus (M) Score**	**Description**
1	Glanular
2	Coronal Sulcus
3	Mid or Distal Shaft
4	Proximal shaft, Penoscrotal
**Shaft (S) Score**	**Description**
1	No chordee
2	Mild (<30°) chordee
3	Moderate (30–60°) chordee
4	Severe (>60°) chordee

**Table 2:** Description of the key variables to be evaluated.

	Variable	Description and Methodology	Tools/Notes	Data Format
**1.**	**Hypospadiac Meatus Location**	Visual classification based on meatus position (glanular to perineal). Photographic documentation required.	Visual inspection; standardized image examples	Categorical: Glanular; Coronal; Distal; Midpenile; Proximal; Scrotal/Perineal.
**2.**	**Urethral Plate Width (UPW)**	Measured using sterile calipers at The narrowest point of the urethral plate; Between the 2 B points.	Two independent evaluators; two measurements each	Continuous (mm).
**3.**	**Urethral Plate Length (UPL)**	Distance from hypospadias meatus to the tip of the glans along the urethral plate.	Ruler or calipers	Continuous (mm).
**4.**	**Urethral Plate Depth/Configuration**	Visual assessment of plate depth: flat, shallow, or deep.	Qualitative assessment	Categorical (deep/flat/shallow).
**5.**	**Glans Width (GW)**	Transverse width at widest point of glans.	Calipers; two evaluators ×2	Continuous (mm).
**6.**	**Glans Depth**	Anteroposterior depth at glans midline.	Calipers; two evaluators ×2	Continuous (mm).
**7.**	**Glans-Urethral Meatus-Shaft (GMS) Score**	Composite score evaluating glans size, meatal location, and shaft curvature.	Utilize standardized scoring sheets WITH at least two independent observers	Continuous (score 3–12 or per scale).
**8.**	**Degree of Chordee**	Assessed by artificial erection test intraoperatively	Photograph (Measured by *Angle Meter 360* Mobile App)	Continuous (degrees).
**9.**	**Stretched Penile Length (SPL)**	From pubic bone to glans tip with gentle stretch.	Calibrated ruler	Continuous (mm).
**10.**	**Urethral Defect Length (UDL)**	Intraoperative measurement of the length of B points to spongiosa bifurcation after degloving	Ruler or calipers	Continuous (mm).
**11.**	**Anogenital Distance (AGD)**	Distance from the center of the anus to the base of the scrotum.	Measured supine with knees flexed; ruler	Continuous (mm).
**12.**	**Plate Objective Scoring Tool (POST)**	Quantitative scoring tool assessing urethral plate quality and shape.	Visual exam + photo documentation	Continuous (score).
**13.**	**Urethral Plate-to-Glans Width Ratio (U/G Ratio)**	Calculated from UPW and GW measurements.	Derived from direct measurements	Continuous.
**14.**	**Urethral Defect Length/SPL**	Calculated from UDL and SPL measurements.	Derived from intraoperative and preoperative measurements	Continuous.

## Abbreviations

AAP	American Academy of Pediatrics
AGD	Anogenital Distance
CI	Confidence Interval
Fr	French (unit of diameter, e.g., for catheters or meatal calibration)
GMS	Glans–Meatus–Shaft Score
GW	Glans Width
HOPE	Hypospadias Objective Penile Evaluation
HOSE	Hypospadias Objective Scoring Evaluation
ICC	Intra-class Correlation Coefficient
IQR	Interquartile Range
OR	Odds Ratio
POST	Plate Objective Scoring Tool
RR	Relative Risk
SD	Standard Deviation
SPL	Stretched Penile Length
UDL	Urethral Defect Length
UPL	Urethral Plate Length
UPW	Urethral Plate Width
UTI	Urinary Tract Infection
U/G Ratio	Urethral Plate Width-to-Glans Width Ratio
